# Turning up the heat: optimizing Bethesda assays for efanesoctocog alfa monitoring

**DOI:** 10.1016/j.rpth.2025.102992

**Published:** 2025-08-05

**Authors:** Agathe Herb, Jordan Wimmer, Dominique Desprez, Olivier Feugeas, Laurent Mauvieux, Laurent Sattler

**Affiliations:** 1 Laboratoire d’Hématologie, Hôpitaux Universitaires de Strasbourg, Strasbourg, France; 2 Centre Régional de Compétences des Maladies Hémorragiques Constitutionnelles, Hôpitaux Universitaires de Strasbourg, Strasbourg, France; 3 Centre National de la Recherche Scientifique (CNRS), Unité Propre de Recherche (UPR) 3572, Immunologie, Immunopathologie & Chimie Thérapeutique, Institut de Biologie Moléculaire et Cellulaire, Strasbourg, France

Efanesoctocog alfa (EFA) is a new therapy for hemophilia A (HA). Unlike earlier generations of recombinant factor (F)VIII, EFA features a unique and substantially larger structure. Indeed, it is composed of a B-domain–deleted, Fc fusion FVIII linked to the D’D3 domain of von Willebrand factor and 2 XTEN polypeptides [1,2]. These structural modifications may impact its laboratory monitoring: while some one-stage assays accurately measure EFA activity, chromogenic assays tend to significantly overestimate it [3]. Additionally, these modifications may also confer increased resistance to heat treatment, a preanalytical step in Bethesda assays designed to eliminate FVIII activity without affecting FVIII inhibitors (A8) [4]. Therefore, the World Federation of Hemophilia recommends performing, prior to testing, a preanalytical heat treatment (PHT) at 56 °C for 30 minutes (T30) [5]. This protocol has been shown to be effective for earlier recombinant FVIII products [6,7]. However, no data are currently available to determine whether this standard duration of T30 of PHT is sufficient to fully degrade EFA.

This study, therefore, aimed to evaluate whether a prolonged PHT is required to ensure complete degradation of EFA, and to assess whether such a modification could alter the titers of potential A8.

This study was approved by the institution’s ethics board (CE-2025-90) and conducted using samples from patients referred to the Hematology Laboratory of Strasbourg’s University Hospital (France). Inclusion criteria were as follows: severe congenital HA treated with EFA or acquired or congenital HA with a positive A8 (≥0.6 Bethesda Units [BU]/mL). Exclusion criteria were as follows: insufficient plasma volume, underaged patients (<18 years old), and treatment with emicizumab.

FVIII activity was measured on a STA-R Max analyzer using CK-Prest, Immunodeficient VIII, and Unicalibrator (all Diagnostica Stago).

To evaluate the heat stability of EFA, samples from persons with congenital HA receiving EFA were tested as follows: first, baseline FVIII activity was measured (T0). Then, samples were subjected to PHT in a water bath at 56 °C for T30, 60 minutes (T60), or 90 minutes (T90). After each time point, samples were centrifuged at 2500 × *g* for 10 minutes, and FVIII activity was measured.

Then, to assess whether prolonged PHT could affect A8 titers, samples from persons with acquired HA were similarly subjected to a PHT in a water bath at 56 °C for T30, T60, or T90, followed by centrifugation at 2500 × *g* for 10 minutes. Bethesda assays were then performed as follows: samples were prediluted using Owren’s Buffer (Diagnostica Stago), then mixed 1:1 with Cryocheck normal pooled plasma (Cryopep), and incubated in a water bath at 37 °C for 2 hours. A control mixture, consisting of 1:1 Cryocheck normal pooled plasma and Owren’s Buffer, was prepared and processed identically. Postincubation, FVIII activity was measured, compared with the control mixture, and A8 titers were calculated as BU per milliliter accordingly [8].

Statistical tests were performed using Prism v6.05 (GraphPad Software). A8 titers at T60 and T90 were compared with those at T30 using a paired Wilcoxon test.

**Figure 1:**
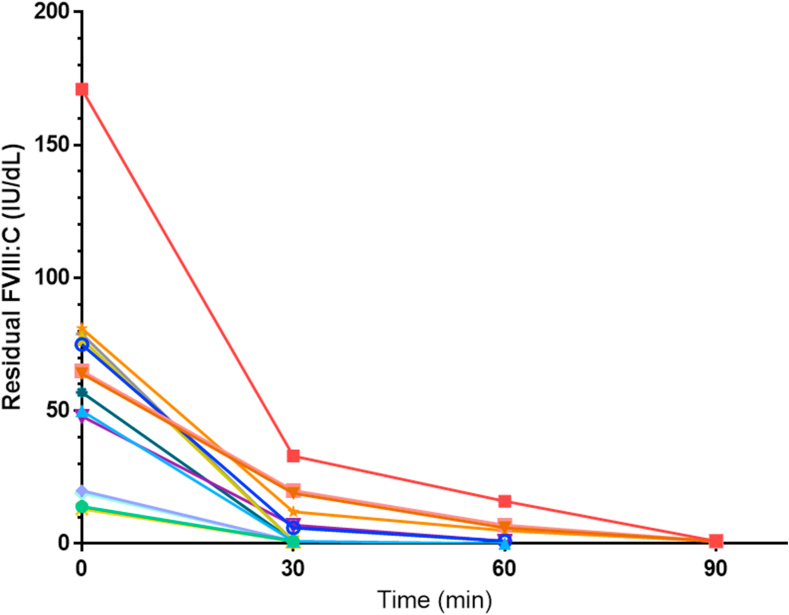
Factor (F)VIII activity (FVIII:C) in samples of patients treated with efanesoctocog alfa before preanalytical heat treatment or after preanalytical heat treatment for 30, 60*,* or 90 minutes. IU, International Units.

A total of 14 samples of patients receiving EFA were included. Mean FVIII (minimum-maximum) activity at T0 and after PHT for T30, T60, and T90 was 59.5 IU/dL (13-171), 7.1 IU/dL (<1-33), 2.5 IU/dL (<1-16), and <1 IU/dL, respectively. Complete degradation of EFA, defined as FVIII activity < 1 IU/dL, was observed in 57% (*n* = 8), 14% (*n* = 2), and 29% (*n* = 4) of samples at T30, T60, and T90, respectively (see Figure 1).

**Figure 2:**
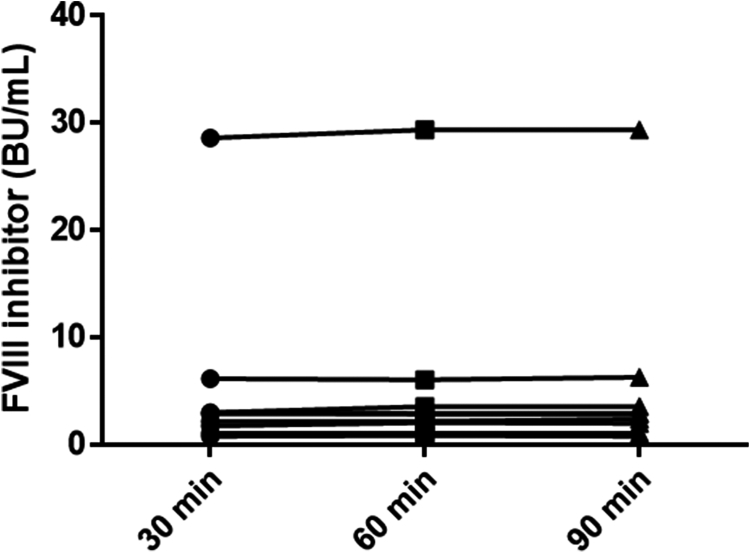
Factor (F)VIII inhibitor titers in samples of patients with FVIII inhibitors after 30, 60, or 90 minutes of preanalytical heat treatment. BU, Bethesda Units.

Additionally, samples of 7 persons with acquired HA and 1 person with congenital mild HA with positive A8 were included. Mean (minimum-maximum) A8 titers at T30, T60, and T90 were 5.5 BU/mL (0.8-28.6), 5.7 BU/mL (0.9-29.4), and 5.7 BU/mL (0.9-29.4), respectively (see Figure 2). There were no significant differences between A8 titers at T30 vs T60 (*P* = .16) or T30 vs T90 (*P* = .06), indicating stable inhibitor measurements across time points.

This study demonstrates that EFA may require extended PHT up to T90 at 56 °C to achieve complete degradation, and that prolonging this step does not affect A8 titers.

First, we showed that only 57% of samples showed complete inactivation of FVIII activity after T30 of PHT, whereas all samples reached undetectable levels after T90 of PHT. This was particularly relevant for samples with T0 FVIII activity ≥ 50 IU/dL. This finding suggests that EFA is more heat-resistant than earlier generations of recombinant FVIII, for whom complete degradation could be achieved after T30 of PHT [6,7].

This has important implications for Bethesda assays, as incomplete degradation of FVIII could result in falsely low or undetectable inhibitor titers [9,10]. Whenever possible, samples should be collected at EFA trough levels to minimize residual FVIII activity.

Second, we demonstrated that extending the PHT duration did not significantly impact A8 titers. Indeed, there were no significant differences between A8 titers after T30, T60, or T90 of PHT at 56 °C, which aligns with previous findings [11].

Our study has several limitations. First, we included a limited number of samples from patients receiving EFA. Second, to assess the heat resistance of A8, we included samples of persons with acquired HA, and only a single sample of a person with mild HA and a positive A8. Given that the characteristics of A8 may vary depending on the type of A8 and the underlying condition [8,12], we cannot exclude the possibility that A8 in congenital HA could be more heat sensitive. However, Boylan and Miller [11] previously demonstrated that A8 in persons with congenital HA was heat-stable after 60 minutes at 56 °C.

In conclusion, these results support the use of extended PHT protocols (ideally, T90) when testing samples of patients treated with EFA without compromising the accuracy of inhibitor detection. Nevertheless, larger-scale studies are required to confirm these results and establish standardized guidelines.
